# The effect of CCL5 on the immune cells infiltration and the prognosis of patients with kidney renal clear cell carcinoma

**DOI:** 10.7150/ijms.51126

**Published:** 2020-10-18

**Authors:** Shuheng Bai, YinYing Wu, Yanli Yan, Haojing Kang, Jiangzhou Zhang, Wen Ma, Ying Gao, Beina Hui, Rong Li, Xiaozhi Zhang, Juan Ren

**Affiliations:** 1 Department of Radiotherapy, Oncology Department, First Affiliated Hospital of Xi'an Jiaotong University, Xi'an, Shaanxi Province, China, 710061;; 2 Department of Chemotherapy, Oncology Department, First Affiliated Hospital of Xi'an Jiaotong University, Xi'an, Shaanxi Province, China, 710061;; 3 Medical School, Xi'an Jiaotong University Xi'an, Shaanxi Province, China, 710061

## Abstract

**Background:** Kidney renal clear cell carcinoma (KIRC) is the most representative subtype of renal cancer. Immune infiltration was associated with the survival time of patients with tumors. C-C chemokine ligand 5 (CCL5) can promote the malignant process of tumor and be related to infiltration immune cells in some cancers, but not reported in KIRC.

**Methods:** The expression profile and clinical data were obtained from The Cancer Genome Atlas (TCGA) database. The correlation between the expression level of CCL5 and clinical features in KIRC was analyzed. Gene Set Enrichment Analysis (GSEA) was utilized to explore the functions and pathways of CCL5 in KIRC. Then, the analysis between the survival and immune infiltration cells was carried out, as well as the non-parametric tests between the CCL5 expression and the ratios of immune infiltration cells.

**Results:** The correlations between the expression levels of CCL5 in KIRC and clinical features including survival time, pathological stage, grade, and status of the patient, have been identified. Meanwhile, GSEA analysis has shown relationships between the expression of CCL5 and immune pathways. The immune infiltrated cells were correlated with the prognosis of KIRC, especially regulatory T cells (Tregs), mast cells, and dendritic cells. And Tregs was associated with the CCL5 expression.

**Conclusion:** The increased expression of CCL5 is related to poor prognosis and clinical features. Meanwhile, CCL5 is related to Tregs ratios and CCL5 may act as a typical chemokine to recruit Tregs in KIRC. CCL5 could be used as a biomarker for the prognosis prediction and a potential therapeutic target for patients with KIRC.

## Introduction

Renal cancer is a common cancer, and its incidence rates in males and females are 5% and 3%, respectively^1^. Kidney renal clear cell carcinoma (KIRC) accounts for 70%-80% of renal cancer, which is the most representative subtype, with the increased incidence rate year by year. Early diagnosis of KIRC is more difficult compared with other cancers, because kidney cancer-related clinical symptoms are atypical. KIRC has poor responses to conventional chemotherapy and radiation therapy^2^. All of these lead to a low five-year survival rate of advanced patients, which is only 10% to 20% 3, 4. Targeted therapy had a positive effect on prolonging the survival time of patients, including VEGF-TKIs, mTOR inhibitors, and monoclonal antibodies as Bevacizumab 5, 6. But the drug-resistance due to long-term use has not been resolved. Novel treatment target is urgently needed. Immune therapy, especially immune checkpoint inhibitors, is a very promising treatment measure in patients with KIRC 7, 8. But not all of the patients can benefit from it. Objective response rate to anti PD-L1 therapy is only around 20%. And patients who had a favorable response to immune checkpoint inhibitors did not show long time remission 7, 9, 10. So, some new immune checkpoints are needed to be discovered urgently.

Chemokines are a type of small secreted protein with a molecular weight of 8-11KDa, composed of 70-125 amino acids. They are classified into CXC, CC, C and CX3C subfamily, according to the position and remaining structure of the first two cysteine residues in the N segment. And The N-terminal of CC subfamily chemokines contains two adjacent Cys residues. CCL5 belongs to the CC family of chemokine and is mainly expressed in T cells, macrophages and some tumor cells 11. Some researche had demonstrated that CCL5 promotes the development of malignant tumors including lung cancer, colorectal cancer, esophageal cancer, breast cancer and so on 12-15. So CCL5 may be utilized as a biomarker to predict prognosis and act as a new target to treat KIRC.

Immune infiltration in the tumor microenvironment has been demonstrated that it is not only associated with the survival time of cancer patients, but also has great potential for cancer treatment^16^, ^17^. Studies revealed some immune infiltration cells, including regulatory T cells, mast cells, CD8+T cells, and dendritic cells, had great effects on KIRC progression^18^-^21^. Moreover, some studies found that CCL5 is related to some immune diseases and regulates immune infiltrating cells which can yield immune escape 22-25. Yet up to now, there are no studies which explore its prognostic value and its relationship with immune infiltration cells in KIRC.

In this study, Relationships between the mRNA expression levels of CCL5 and the clinical-pathological features, infiltration immune cell landscape of KIRC patients were analyzed based on TCGA database and bioinformatics analysis including CIBERSORT and GSEA. The purpose of this study was to evaluate the prognostic value of CCL5 and to explore the potential relationship between CCLR and infiltration immune cells in KIRC patients.

## Methods

### Data acquiring and related bioinformatics analysis

The transcriptome profiling data about Kidney Renal Clear Cell Carcinoma and corresponding clinical information were obtained from TCGA official website (https://cancergenome.nih.gov/), which is a public database demonstrated major cancer related genomic alterations 26. Gene expression data (level 3) based on HTSeq-FPKM workflow, which including 539 tumor samples and 72 control samples (normal tissue adjacent to tumor in the same patient), and corresponding clinical data were obtained. Then boxplots were used to show the differences of genes expression between normal groups and tumor groups, and some clinical variables (stage, age, gender, pathologic grade, TNM). Finally, the survival curve (K-M) was applied to explore the relationships between CCL5 expression level and the overall survival time of patients with KIRC.

### Gene set enrichment analysis

Gene set enrichment analysis (GSEA) is a method to determine that if predefined sets of genes are differentially expressed in different phenotypes. Predefined gene sets may be genes in a known metabolic pathway, located in the same cytogenetic band, sharing the same Gene Ontology category, or any user-defined set^27^. In this study, GSEA was carried out to explore the significant signal pathways related to CCL5 groups. Gene set permutations were performed 1000 times for each analysis. The c2.cp.kegg.v7.0.symbols.gmt data set was obtained from the GSEA website. The expression level of CCL5 was used as a phenotype label. The p value represents the credibility of the enrichment result, q value is the p value after multiple hypothesis testing correction. The nominal p value and normalized enrichment score (NES) were used to sort the pathways enriched in each phenotype.

### Assessment of immune infiltration

CIBERSORT (http://cibersort.stanford.edu/) is an analytical tool to provide an estimation of the abundances of immune cells in mixed cell populations, using gene expression data 28. It was used to evaluate the relative ratios of 22 types of infiltration immune cells in each cancer example, which including M1 macrophages, M2 macrophages, M0 macrophages, T follicular helper cells, resting memory CD4+ T cells, activated memory CD4+ T cells, γδT cells, CD8+ T cells, Tregs CD4+ T cells, naive CD4+ T cells, resting natural killer (NK) cells, activated NK cells, resting mast cells, activated mast cells, memory B cells, resting dendritic cells (DC), activated DC, naïve B cells, monocytes, plasma cells, neutrophils and eosinophils 29. *p*‑value of CIBERSORT, which can evaluate the statistical significance of the deconvolution consequences, was used as set *p*<0.05.

### Identification of the relationship between the profile of CCL5 and immune infiltration cells

Mann-Whitney test was used to evaluate the relationships between the expression of CCL5 and some types of immune infiltration cells which had effects on the overall survival time. The significant fitting accuracy was set as *p*<0.01.

Gene Expression Profiling Interactive Analysis (GEPIA) is a web-based tool to deliver fast and customized functionalities based on TCGA and GTEx data. It provides key interactive and customizable functions including differential expression analysis, profiling plotting, and the correlation analysis^30^. The “correlation” module was utilized to confirm the relationships between CCL5 expression and the possible gene markers of tumor-infiltrating immune cells which had been selected through the above process 16.

### Statistical analysis

All statistical analyses were conducted by using R (v.3.6.1), GraphPad_Prism (V 8.0) and SPSS (V 25). Non-normally distributed data used Wilcoxon test and Chi-square test. Kaplan-Meier method was utilized to analyze the overall survival. Mann-Whitney test (U-test) was used to analyze the correlation between two groups.

## Results

### The relationship between the mRNA expression levels of CCL5 and the clinical features of patients with KIRC

**Fig 1:**
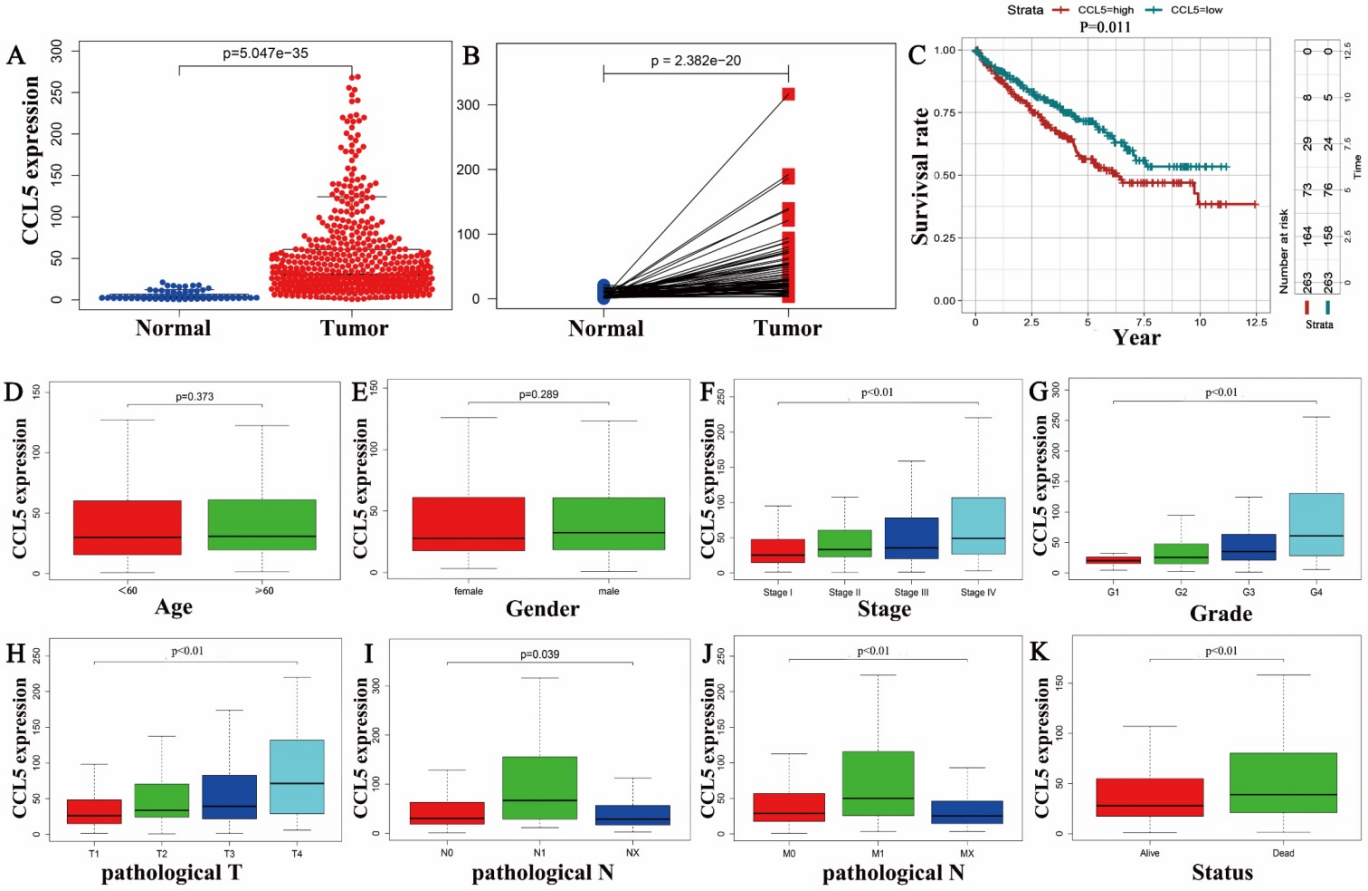
** The relationship between CCL5 and the clinical features.** (A) Different expression profiles of CCL5 between 539 KIRC tissues and 72 normal tissues. (B) Different expression profiles of CCL5 between 72 KIRC tissues and matched normal tissue from the same patients. (C) Effect of CCL5 expression on OS in all patients, *p*=0.011. (D-K) Relationships between CCL5 expression and clinical characters of patients with KIRC including the age, gender, stage, grade, pathologic T, pathologic N, pathologic M, and status.

The CCL5 mRNA levels of 539 KIRC tissues were significantly higher than that of 72 normal tissues (P<0.01) as shown in Figure 1A, and consistent results were showed in the 72 matched tissue samples from the KIRC patients (*p*<0.001) (Figure 1B). In the group of KIRC, CCL5 expression ranged from 0.87 to 269.103, and the median expression was 30.5, which was a criterion to define low and high expression groups. Survival analysis showed that the patients with high expression levels of CCL5 exhibited significantly shorter overall survival time (OS) (p=0.011) than the patients with low CCL5 expression levels, shown in Figure 1C.

Then, boxplots were performed to show the relationships between the expression of CCL5 and clinical characters including the age (*p*=0.373), gender (*p*=0.289), stage (*p*<0.01), pathologic T (*p*<0.01), pathologic N (*p*=0.039), pathologic M (*p*<0.01), grade (*p*<0.01), status (*p*<0.01), shown in Fig 1D-K.

High expression levels of CCL5 of patients with KIRC were related to some clinical characters as shown in Table 1. Stage, pathological T stage, pathological M stage, the status of patients, tumor pathological grade were all significantly related with CCL5 high expression in patients with KIRC (all meet *p*<0.01); However, some clinical characters including age, gender, and pathologic N stage were not associated with the expression levels of CCL5 in patients with KIRC.

### KEGG pathways related to CCL5

To identify the CCL5 related signaling transduction pathways in KIRC, GSEA analysis was performed between the data of high CCL5 expression levels of and that of low CCL5 expression. GSEA revealed significant differences in the enrichment of the KEGG Collection between CCL5 high expression group and low CCL5 expression groups.

**Fig 2:**
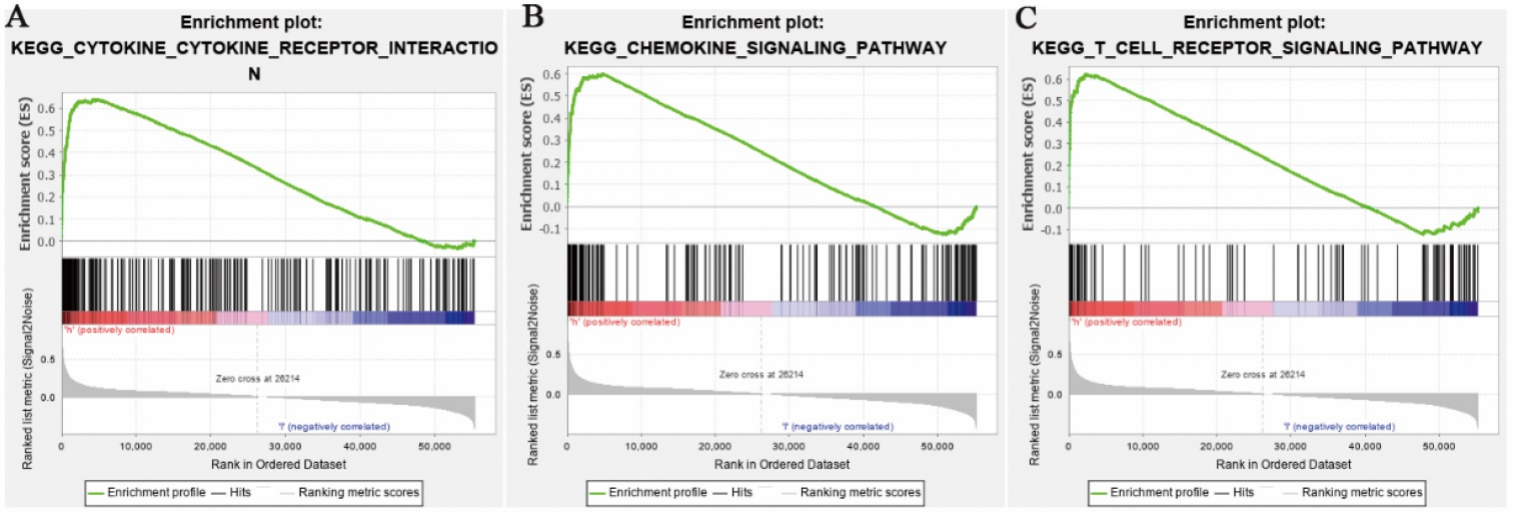
** Enrichment plots from GSEA**. (A) Chemokine signaling pathway. (B) Cytokine-cytokine receptor interaction pathway. (C) T cell receptor signaling pathway separately.

Relied on the false discovery rate (FDR) and the normalized enrichment score (NES), top 20 signaling transduction pathways which were satisfied with *p*<0.01 were ranked by NES, as shown in Table 2. It contained chemokine signaling pathways. The top 5 pathways were cytokine-cytokine receptor interaction, autoimmune thyroid disease, antigen processing and antigen presentation, natural killer cell mediated cytotoxicity, and immune network for IgA production. Among these pathways, some pathways were related to immune response. So, a speculation that the expression levels of CCL5 may play an important role in immune response in patients with KIRC was raised. Figure 2 showed chemokine signaling pathway, cytokine-cytokine receptor interaction pathway and T cell receptor signaling pathway analyzed by GSEA.

### Overview of immune infiltration in RCC and normal samples

**Fig 3:**
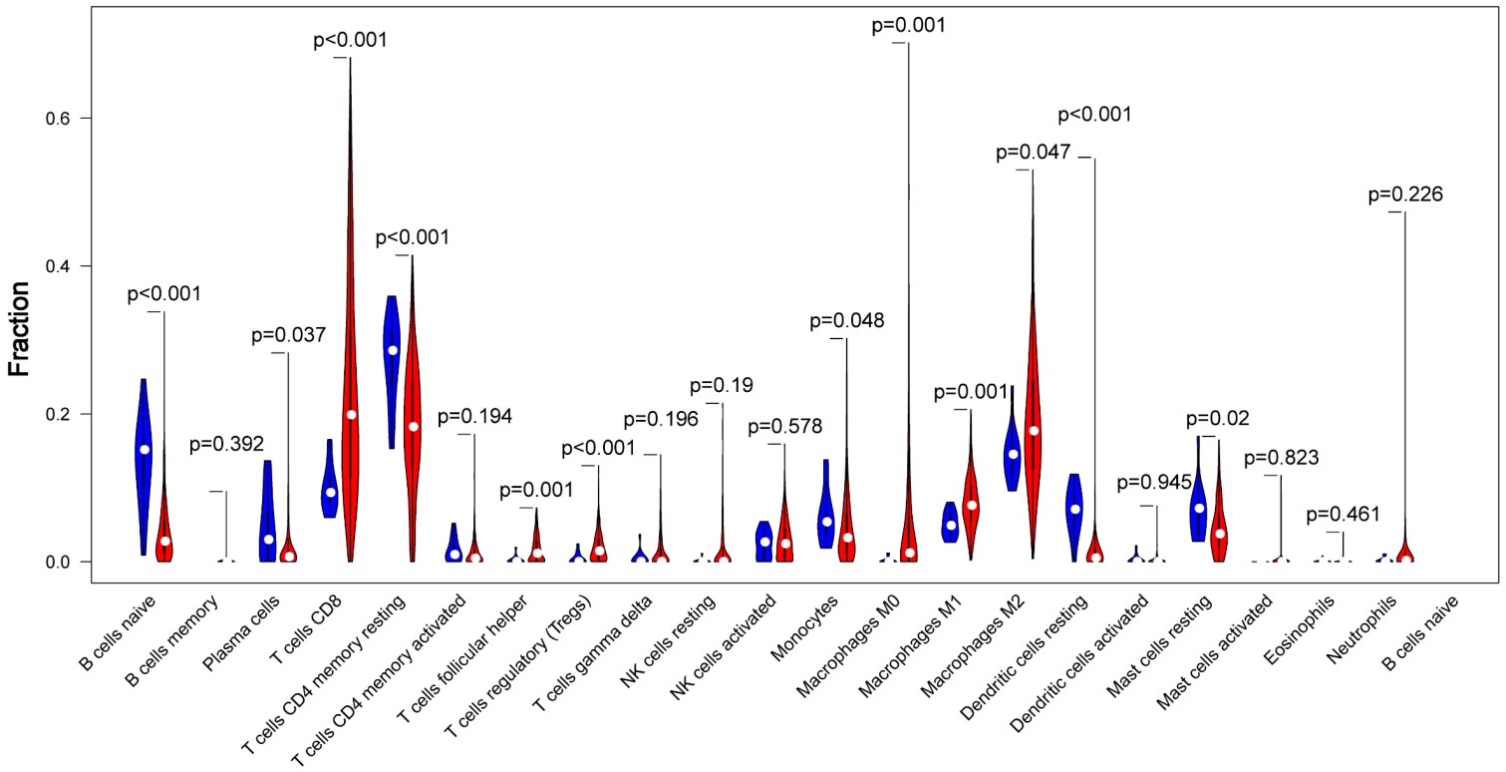
** The proportions difference of all 22 types of immune infiltration cells between tumor tissue and normal tissue.** Comparisons of 22 immune cells between tumor (red parts) and normal (blue parts) tissues showed that naive B cell, CD4+ memory resting T cells, resting Dendritic cells were lower in the tumor group, and CD8+ T cells, Tregs were higher in tumor group, compared with the normal group (p<0.01).

CIBERSORT, which provides an estimation of the abundance of immune cells in a mixed cell populations, was utilized to assess the proportion of 22 immune cell types in different samples. The proportions difference of all 22 types of immune infiltration cells between tumor tissue and normal tissue was analyzed. The results illustrated that 5 types of immune cells were significantly different between the two groups (*p*<0.01) as shown in Fig 3. Among these cells, naive B cell, CD4+ memory resting T cells, resting Dendritic cells were significantly lower in tumor group compared with the normal group, While CD8+ T cells, Tregs were significantly higher in tumor group (*p*<0.01).

### Overall survival time of immune infiltration cells in KIRC

**Fig 4:**
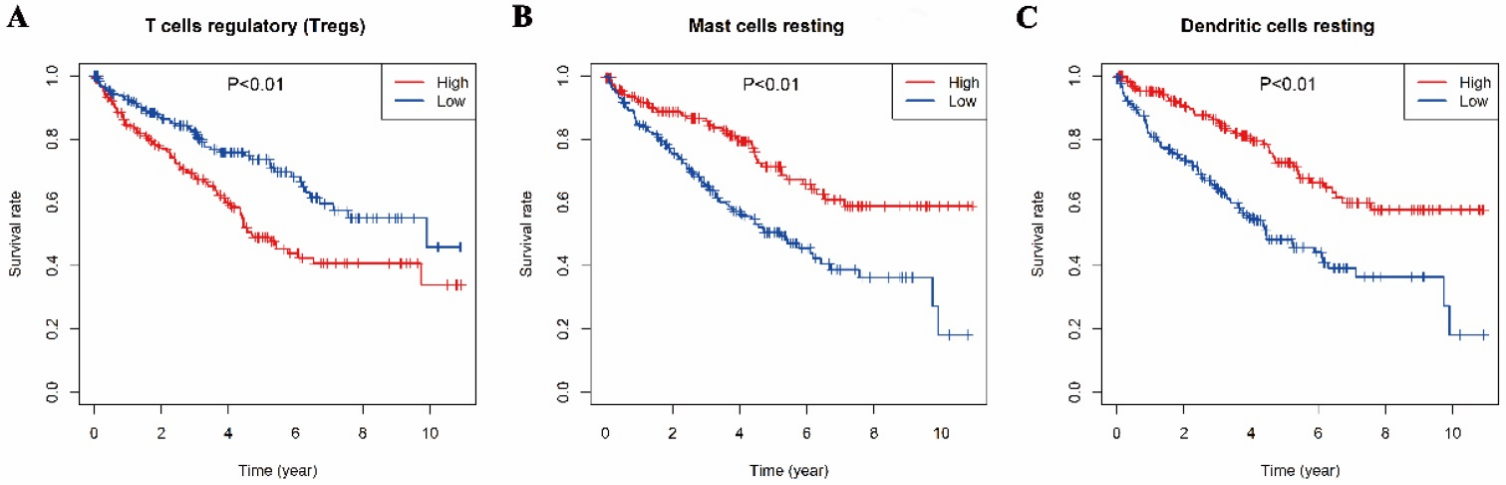
Relationships between the Overall survival time and three types of immune infiltration cells. A, regulatory T cells. B, resting mast cells. C, resting dendritic cell

The relationships between the 22 types of immune cells and the overall survival time of patients were analyzed by survival analysis (K-M). It was found that three types of immune cells had a significant impact on the overall survival time. As showed in Fig 4, T cells regulatory, mast resting cells and dendritic resting cells had significant effects on the overall survival time (*p*<0.01). Regulatory T cells had a negative regulation on the survival time of patients with KIRC which is consistent with their function, but resting mast cells and resting dendritic cells had positive effects on it.

### Relationship between CCL5 expression and immune infiltration cells

**Fig 5:**
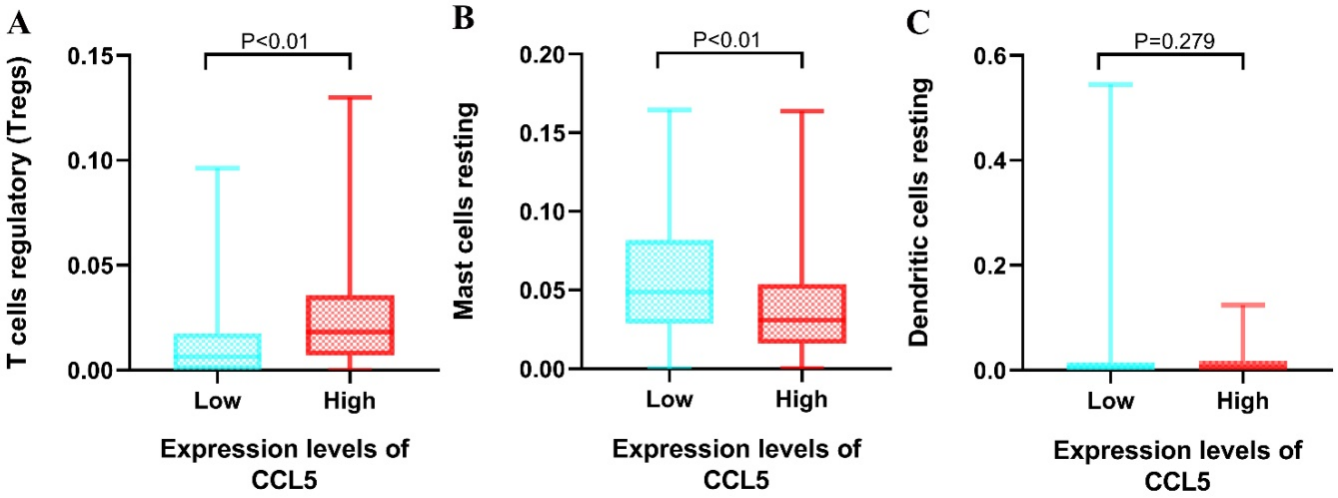
** Relationship between CCL5 expression and immune infiltration cells.** Mann-Whitney test was utilized, and the low and high expression groups of CCL5 were grouped by CCL5 median expression (30.5). A, regulatory T cells. B, resting mast cells. C, resting dendritic cell.

Regulatory T cells, resting Mast cells and resting dendritic cells had shown significant effects on the OS. Then, non-parametric tests including Kruskal-Walls test, Mann-Whitney test (U-test) and independent sample median test were utilized to explore the relationships between the 3 types of immune infiltration cells and the expression levels of CCL5. The group of high expression levels of CCL5 had positive effects on the proportions of regulatory T cells (median 0.183 *vs* 0.064, *p*<0.01). The significant correlation between the expression levels of CCL5 and the proportions of resting mast cells were found (median 0.308 *vs* 0.049, *p*<0.01) shown in Fig 5. It was found that the proportion of resting dendritic cells was not influenced by the expression levels of CCL5.

Then, “correlation” module (Spearman test) of GEPIA was used to confirm the result. The gene biomarkers of mast cells, including CPA3, TPSB2, TPSAB1, MS4A2, HDC, were not correlated with CCL5. But the gene biomarkers of Tregs, including CD2, CD25, Foxp3, CTLA-4, were correlated with CCL5 significantly (*p*<0.01), shown in Table 3. So, CCL5 may play an important role in regulating the abundance of Tregs in KIRC.

## Discussion

In this study, the correlation between the expression levels of CCL5 in KIRC and some clinical features including the survival time, pathological stage, and so on, was analyzed to explore the value of CCL5 as a tumor biomarker in KIRC. Meanwhile, KEGG pathways analysis for CCL5 showed an interesting relationship between the expression of CCL5 and some immune signaling transduction pathways, such as T cells receptor pathway. CIBERSORT was utilized to explore the relationship between the difference of ratios of 22 types of immune infiltration cells and the different expression levels of CCL5 in KIRC. The relationships were verified by the “correlation” module of GEPIA. The results showed that the expression of CCL5 is related to regulatory T cells, which led to poor survival time in patients with KIRC.

Kidney Renal Clear Cell Carcinoma (KIRC) is the most representative subtype of kidney cancers. However, the clinical treatment effect is poor, and the 5-year survival of patients with the advanced stage is as low as 12%^6^. So, identifying more biomarkers for diagnosis and prognosis is important and urgent. CCL5 is widely secreted by natural killer cells, T cells, fibroblasts, epithelial cells, platelets, and some certain types of tumor cells 31, 32. It has been demonstrated as a biomarker for the prognosis in esophageal cancer, colorectal cancer, breast cancer and so on 33-35. But in kidney renal clear cell carcinoma, there was only one study which implied that CCL5 may be a crucial potential gene associated with carcinogenesis of KIRC based on the literature review 36. This present study found that CCL5 mRNA was significantly overexpressed in KIRC tissues compared with normal tissues adjacent to cancer. This study also revealed significant connection between the expression levels of CCL5 and disease stage, pathological grade, and pathological TNM stage. In addition, survival analysis also revealed that the high expression level of CCL5 induced a poor survival time compared with the low expression group. These results indicate that CCL5 gene plays an important role in the development of KIRC.

Multiple studies have confirmed that chemokines, which contain 4 subgroups CXC, CC, CX3C and C, play important roles in regulating tumor microenvironment. These chemokines including CCL5 can bind to seven-transmembrane G protein receptors on the target cell membrane, thereby activating downstream effector molecules and promoting the malignant process of tumors. CCL5 promotes tumor cell proliferation through the mTOR pathway or increases glucose uptake. It can also indirectly promote tumor cell proliferation by recruiting macrophages, fibroblasts, T cells, etc 37, 38. At the same time, CCL5 can promote angiogenesis through promoting endothelial cell migration, neovascularization, and the secretion of vascular endothelial growth factor (VEGF) 39-41. In addition, it plays an important role in tumor cell migration through upregulating MMP2 and MMP9 expression or activating integrin 42-44.

In this study, KEGG pathways selected by NES scores from GSEA results contained chemokine signaling pathway and cytokine-cytokine receptor interaction, which may also verify the functions of CCL5. Meanwhile, many signaling transduction pathways related to immune functions, such as T cell receptor pathways, were contained in these KEGG pathways. Studies had revealed that CCL5 promoted tumor migration and invasion through recruiting M2 type macrophages (TAMs) in breast cancer 32. Meanwhile, Tregs had been found that it was recruited by CCL5-CCR5 axis in ovarian cancer 45. CCL5 may have effects on tumor infiltration cells. So, CIBERSORT was utilized to explore tumor infiltration immune cells in KIRC. Tumor infiltration immune cells are a major component of the tumor microenvironment and are closely related to tumor cell proliferation, treatment response and prognosis 46, 47. Different proportions of Regulatory T cells (Tregs) including naive B cell, resting CD4+ T cells memory, resting dendritic cells, CD8+ T cells were identified between tumor tissues and normal tissue. And among these five types of tumor infiltration immune cells, Tregs was the only one, which not only significantly had effects on survival time but also was significantly related to the expression levels of CCL5 in KIRC.

Tregs are a group of lymphocytes which negatively regulate the body's immune response and play an important role in maintaining self-tolerance and avoiding the immune response from self-damage. It is simply divided into two types by different sources, one is natural Treg (nTreg) which is derived from thymus and mostly expressed FOXP3, another is inducible Treg (iTreg) which is induced peripherally^48^. Tregs have been studied because of its function of suppressing the bodies' anti-tumor immune response 49. At present, it is thought that the immunosuppressive effect is exerted mainly through the following ways. Firstly, Tregs are activated by T cell receptor (TCR) to directly inhibit the formation of effector T cells. Secondly, Treg inhibit the activity of immune cells through cross-linking between cell surface cytotoxic T lymphocyte-associated antigen-4 (cTLA-4) and antigen-presenting cells (APC). Thirdly, Tregs secrete cytokines such as IL-10 and TGF-B which can inhibit the function of effector T cells 50, 51. Some research reported that an increased number of Tregs no matter in peripheral blood or in tumors have a negative correlation with survival time in KIRC 52-54. Some studies utilized various methods to deplete Treg cells or control Treg cell function and these methods were effective in treating tumors by increasing immune ability of anti-tumor^55^, ^56^. But the lack of special targets for depletion and functional impairment of Treg cells is a vast obstacle to overcome^57^.

CCL5 acts as an important factor in recruiting Tregs and assisting Tregs to implement its function suggested by some studies. A research had demonstrated that tumor-infiltrating Tregs were increased by doing intratumoral injection of CCL4 or CCL5 in mice models. It also shown an interesting result that tumor-infiltrating MO-MDSCs (myeloid-derived suppressor cells) produce high levels of the CCL3, CCL4, and CCL5 which is CCR5 ligands, and directly attract Tregs in a CCR5-dependent manner 58. Meanwhile, a study identified that zoledronic acid can significantly affect the interaction between Tregs and cancer cells by reducing the expression of CCL5 and CCL2^59^. Furthermore, Tregs, which were recruited by CCL5, promoted the invasion of ovarian cancer cells through matrix metalloproteinase-9 (MMP9), which enhanced the degradation of the extracellular matrix and enabled the invasion of tumor cells, also been identified 45, 60. So, this present study demonstrated that a higher expression level of CCL5 is associated with the more abundance of Tregs in KIRC. CCL5 may act as a typical chemokine to recruit Tregs to mediated immunosuppression and lead to immune escape in KIRC. Furthermore, down regulation of CCL5 in KIRC may improve the immune effect by decreasing recruitment of Tregs. CCL5 may act as a target of immune therapy in the future.

In summary, the increased expression of CCL5 is related to poor prognosis and some clinical features including TNM stage, pathological grade. So CCL5 could act as a biomarker for the prognosis and potential therapy target for patients with KIRC. Meanwhile, the poor prognosis may be caused by the function of CCL5 to recruit more Tregs which induced the immune suppression in patients with KIRC.

All these results were based on a series of bioinformatics algorithms and databases. Experiments, as immunohistochemistry, are needed to confirm these results.

### Availability of data and materials

The datasets used and analyzed during the current study are available from the TCGA database.

### Funding

This manuscript is supported by the National Natural Science Foundations of China (Juan Ren, 81772793/H1621, Juan Ren, 31201060/C0709; Juan Ren, 30973175/H1621; and Juan Ren, 81772793/H1621); Supported by Scientific and Technological Research Foundation of Shaan'xi Province (Juan Ren, 2020JM-368); Supported by Program for New Century Excellent Talents in University (Juan Ren, NCET-12-0440); The Fundamental Research Funds for the Central Universities (Juan Ren, 2012).

### Author Contributions

JR conceived and supervised the study; JR, SHB, YYW, YYL, HJK, JZZ, WM, YG, RL, and BNH analyzed data; JR and SHB wrote the manuscript; JR, SHB and XZZ made manuscript revisions. All authors have read and approved the final version of this submission.

## Figures and Tables

**Table 1:** The clinical characteristics of KIRC patients between high expression of CCL5 group and low expression group.

Clinical characteristics	Variable	No. patients	CCL5 expression		χ^2^	P
			low(<30.5)	high(≥30.5)		
**Age**	<60	246	124	122	0.03	0.862
	≥60	284	141	143		
**Gender**	female	186	101	85	2.12	0.154
	male	344	164	180		
**Status**	alive	357	196	161	10.51	<0.01
	dead	173	69	104		
**Grade**	G1	14	11	3	35.46	<0.01
	G2	227	139	88		
	G3	206	87	119		
	G4	75	21	54		
**pathologic T**	T1	271	162	109	21.28	<0.01
	T2	69	28	41		
	T3	179	71	108		
	T4	11	4	7		
**pathologic N**	N0	239	119	120	4.29	0.117
	N1	16	4	12		
	NX	275	142	133		
**pathologic M**	M0	421	221	200	10.25	<0.01
	M1	78	26	52		
	MX	30	17	13		
**pathologic Stage**	stage I	265	160	105	27.77	<0.01
	stage II	57	25	32		
	stage III	123	52	71		
	stage IV	82	27	55		

**Table 2:** Gene sets enriched in the samples of KIRC in the high expression of CCL5 group.

Gene set name	SIZE	ES	NES	NOM p-val	FDR q-val
**KEGG_CYTOKINE_CYTOKINE_RECEPTOR_INTERACTION**	264	0.64015	2.7222579	0	0
**KEGG_AUTOIMMUNE_THYROID_DISEASE**	50	0.90781	2.7134721	0	0
**KEGG_ANTIGEN_PROCESSING_AND_PRESENTATION**	81	0.82137	2.6439135	0	0
**KEGG_NATURAL_KILLER_CELL_MEDIATED_CYTOTOXICITY**	132	0.69761	2.6304462	0	0
**KEGG_INTESTINAL_IMMUNE_NETWORK_FOR_IGA_PRODUCTION**	46	0.88617	2.6202402	0	0
**KEGG_CELL_ADHESION_MOLECULES_CAMS**	131	0.69955	2.5745416	0	0
**KEGG_VIRAL_MYOCARDITIS**	68	0.77404	2.4800615	0	6.15E-05
**KEGG_HEMATOPOIETIC_CELL_LINEAGE**	85	0.69017	2.4422312	0	1.08E-04
**KEGG_TYPE_I_DIABETES_MELLITUS**	41	0.9242	2.4161355	0	2.39E-04
**KEGG_PRIMARY_IMMUNODEFICIENCY**	35	0.87674	2.3683138	0	3.42E-04
**KEGG_CYTOSOLIC_DNA_SENSING_PATHWAY**	55	0.64324	2.3671436	0	3.11E-04
**KEGG_ASTHMA**	28	0.86854	2.3409052	0	3.86E-04
**KEGG_CHEMOKINE_SIGNALING_PATHWAY**	188	0.59863	2.278743	0.001934236	6.73E-04
**KEGG_ALLOGRAFT_REJECTION**	35	0.945	2.2597005	0	6.87E-04
**KEGG_LEISHMANIA_INFECTION**	70	0.75627	2.2485812	0.001992032	6.64E-04
**KEGG_GRAFT_VERSUS_HOST_DISEASE**	37	0.92916	2.2390857	0	6.98E-04
**KEGG_T_CELL_RECEPTOR_SIGNALING_PATHWAY**	108	0.6243	2.190299	0.001941748	0.00127215
**KEGG_SYSTEMIC_LUPUS_ERYTHEMATOSUS**	135	0.72048	2.1846733	0	0.001251814
**KEGG_JAK_STAT_SIGNALING_PATHWAY**	155	0.48635	2.0852885	0.001912046	0.003184194

**Table 3:** The results of the correlation between CCL5 and gene markers of mast cells and Tregs by the “correlation” module of GEPIA.

	Gene markers	P	R
**Mast cells**	CPA3	0.72	0.016
	TPSB2	0.11	0.07
	TPSAB1	0.085	0.076
	MS4A2	0.58	0.024
	HDC	0.57	0.025
			
**Tregs**	CD4	<0.01	0.88
	CD25	<0.01	0.27
	Foxp3	<0.01	0.62
	CTLA-4	<0.01	0.64
